# Feasibility and Preliminary Dietary Outcomes of the Smart Family Lifestyle Counseling Intervention in Greek Primary Care: A Single-Arm Pilot Study from Health4Eukids

**DOI:** 10.3390/nu18121848

**Published:** 2026-06-08

**Authors:** Emmanuella Magriplis, Niki Myrintzou, Ios-Ioanna Desli, Eleni Papachatzi, Apostolos Vantarakis

**Affiliations:** 1 Laboratory of Dietetics & Quality of Life, Department of Food Science and Human Nutrition, Agricultural University of Athens, 11855 Athens, Greece; nmyrintzou@aua.gr; 2 Laboratory of Public Health, Epidemiology and Quality of Life, Medical School, University of Patras, 26540 Patras, Greece; avanta@upatras.gr; 3 Laboratory of Hygiene, Social & Preventive Medicine and Medical Statistics, School of Medicine, Aristotle University of Thessaloniki, 54124 Thessaloniki, Greece; iosdesli@gmail.com; 4 Department of Pediatrics, University General Hospital of Patras, 26504 Patras, Greece; elepapach@upatras.gr

**Keywords:** childhood obesity, Mediterranean diet, parental perception, dietary behavior, family-based intervention, primary care, pilot study, Smart Family, feasibility

## Abstract

Background: Childhood obesity is a complex public health issue in which parental perceptions and family dietary behaviors are pivotal. This study assessed the feasibility of the Smart Family lifestyle counseling intervention in Greek primary care. It explored changes in children’s dietary behaviors relative to parental weight perception and Mediterranean diet adherence. Methods: A single-arm pretest–posttest pilot study was conducted in Patras, Greece, from Health4EUKids Joint Action. The intervention consisted of four monthly face-to-face counseling sessions using the Smart Family methodology. In total, 49 parent–child dyads (aged 2–12 years) completed the program. Data collection included child anthropometric measurements, validated food frequency questionnaires, parental perception of child weight status, and parental Mediterranean diet adherence. Results: Parents who underestimated their child’s weight status had significantly higher Mediterranean diet scores than those who overestimated (*p* = 0.032); those with low adherence tended to overestimate and those with moderate adherence to underestimate. The largest reduction was observed for sweets and desserts (median −2.35 servings/week), with significant reductions in sugar-sweetened beverages, grains and cereals, whole wheat products, and dairy. Fish and vegetable intake increased significantly, but fruit intake did not change. Changes in fast food and red meat differed significantly across Mediterranean diet score tertiles, with larger decreases in the lower tertiles. Conclusions: Smart Family counseling was feasible to deliver through trained healthcare professionals in Greek primary care over four months, with reductions in selected discretionary foods observed alongside the intervention. Parental weight perception and Mediterranean diet adherence emerged as potential barriers to change although the findings are exploratory and require confirmation in a future controlled trial.

## 1. Introduction

Childhood obesity has become one of the most critical global public health concerns, with prevalence increasing over the past few decades. According to the World Health Organization (WHO), approximately one in three European children has overweight or obesity, increasing their risk of adverse physical and psychosocial health outcomes [1]. The etiology of childhood obesity is multifactorial, involving genetic predisposition, environmental influences, and behavioral patterns [2]. Environmental and behavioral factors are major contributors to the childhood obesity epidemic and are potentially modifiable, with the family environment playing a central role in shaping children’s lifestyle behaviors. Over recent decades, childhood diets across Europe have shifted towards lower nutrient density dietary patterns, with higher intakes of ultra-processed foods, added sugars, and saturated fats, alongside lower consumption of fruits, vegetables, legumes, and whole grains [3]. Children’s dietary choices and lifestyle behaviors are strongly influenced by parental perceptions and behaviors, as parents shape food availability, meal structure, food-related norms, and broader health attitudes within the household [4]. Overall, healthier habits adopted by the whole family have been associated with improved dietary quality and weight-related outcomes in children [5]. A major barrier to childhood obesity prevention is that approximately 50–85% of parents do not recognize overweight or obesity in their children, with accurate recognition becoming more likely only at higher levels of obesity severity [6,7,8], with accurate identification increasing mainly at the high end of obesity ranges [9]. Accurate perception has been associated with children’s weight status, sex, parental education [10], age [6], and parental perceived unhealthy dietary habits of their children [11]. Family-based interventions are therefore recognized as an important component of childhood obesity prevention and treatment strategies [12,13]. However, early intervention can be challenging when parents do not perceive their child’s weight or diet as requiring change. Given these challenges, intervention approaches need to consider not only children’s dietary behaviors but also parental perceptions and family dietary patterns, which may influence engagement with counseling and subsequent behavior change.

The effectiveness of childhood obesity interventions has been examined in systematic reviews [14,15] and parental role modeling has been identified as a potentially more constructive approach for improving children’s diets than overt dietary control [4,16]. Systematic reviews of randomized clinical trials have reported that diet interventions, with or without physical activity components, have small to moderate effects on childhood obesity prevention when compared with control conditions [14,15]. The Special Turku Coronary Risk Factor Intervention Project (STRIP), which began in Turku, Finland, in 1989 and followed children from infancy for 20 years, demonstrated favorable effects on several cardiovascular risk markers and was associated with a lower prevalence of overweight and obesity among girls [17,18,19]. The STRIP used individualized dietary counseling delivered twice yearly and based on Nordic Nutrition Recommendations. Its findings suggest that early, sustained, individualized counseling may be more effective than short-term prescriptive approaches for strengthening parental engagement with healthy eating and food choice behaviors [16]. The Smart Family methodology was developed from this broader tradition of structured, family-centered lifestyle counseling and has been recognized by the European Commission as a best practice for childhood obesity prevention. However, despite evidence supporting individualized nutrition and lifestyle counseling, few studies have examined how parental weight perception accuracy and parental dietary quality may influence engagement with family-based nutrition interventions, even when evidence-informed approaches are used. The present pilot was conducted within Health4EUKids, a European Joint Action aiming to support the implementation of evidence-informed best practices for childhood obesity prevention across participating countries. In Greece, the project provided the framework for adapting and piloting the Smart Family counseling approach in primary care, with the objective of assessing its feasibility and generating preliminary evidence to inform future controlled implementation. The Smart Family model was selected because it provides a structured yet flexible counseling framework that can be delivered by trained healthcare professionals in primary care. Its emphasis on positive family interaction, achievable goal setting, role modeling, and non-stigmatizing discussion of weight-related behaviors was considered particularly appropriate for early childhood obesity prevention, where parental engagement and sensitivity around child weight status are central implementation challenges.

The aim of the present study was to assess the feasibility of implementing the Smart Family lifestyle counseling intervention in Greek primary care and to explore preliminary changes in children’s dietary behaviors over four months. We also examined whether parental perception of child weight status and parental Mediterranean diet adherence were associated with dietary change patterns, with the aim of informing future controlled family-based obesity prevention interventions.

## 2. Materials and Methods

### 2.1. Study Design

The Health4EUkids project is a European intervention program aiming to prevent childhood obesity through the adaptation and implementation of best practices promoting healthy eating and physical activity. The program commenced in 2023 in Patras, the third largest city of Greece and the biggest capital of the 6th Health Administration Authority (Western Greece).

This was a single-arm pretest–posttest pilot implementation study conducted within the Health4EUkids project. It was implemented in the city of Patras and its nearby suburban areas as part of the European Health4EUkids initiative. Patras was selected as the implementation site because regional surveillance data indicated that Western Greece had a high prevalence of childhood overweight and obesity, making it a priority setting for piloting evidence-informed prevention interventions [20]. The intervention was based on the Finnish Smart Family model, which promotes lifestyle change through positive, family-centered counseling. Structured monthly sessions were conducted by healthcare professionals who were trained in the Smart Family methodology. Written informed consent was obtained from all parents before their participation, and granted ethical sanction (Health4EUkids program; grant number 101082462). The study was conducted in accordance with the World Medical Association Declaration of Helsinki and included two phases: healthcare professional training and intervention implementation. This single-arm pretest–posttest design was appropriate for assessing implementation feasibility and describing preliminary within-participant changes over the four-month pilot period. However, because the study did not include a concurrent control group, observed changes cannot be attributed causally to the intervention and should be interpreted as exploratory.

The first phase involved training healthcare professionals, and the second phase involved implementation of the intervention with parent–child dyads. During implementation, all study data were anonymized; identifying information was retained only by the healthcare professional who recruited each dyad. In the training phase, an official communication was sent by the Greek Ministry of Health’s 6th Health Region Administration to healthcare professionals working in health centers and local health units in Patras. The communication described the program objectives and invited professionals to attend two voluntary two-hour virtual training sessions on the Smart Family methodology and tools to be used during implementation. Smart Family tools developed by THL were adapted for the Greek population, following formal written authorization from THL as a WP6 coordinator (part of the Joint EU Health4EUKids program agreement). Training covered use of the Smart Family Health Card, family goal setting, and role-playing exercises to practice motivational counseling skills. Special attention was given to approaching sensitive issues such as childhood overweight in a supportive and empathetic manner. Professionals were also given access to the program’s website through which they could download session materials, handouts, and multimedia resources embedded directly into family counseling (https://healthysmartfamilies.gr/). Trained healthcare professionals were invited to enroll parent–child dyads into the four-session intervention after obtaining parental informed consent. Of the 14 professionals who completed training, 8 participated in the implementation phase.

Before implementation began, trained healthcare professionals attended a kick-off meeting during which the program was reviewed and materials for the first parent–child session were distributed. The intervention consisted of four monthly sessions, with specific guidelines and materials provided to healthcare professionals for each session. Materials for each subsequent session were distributed after completion and submission of documentation from at least one parent–child dyad to the 6th Health Region Administration representative. A summary of the topics and activities per session is included in the Supplementary Materials (Table S1). Feasibility was examined as deliverability of the Smart Family methodology in Greek primary care among included parent–child dyads, completion of the four-session counseling protocol, availability of outcome data, implementation feedback from trained healthcare professionals, and procedures used to support adherence to the counseling protocol. Recruitment rate, refusal rate, number approached, reasons for refusal, and population reach were not predefined feasibility outcomes and were not systematically collected in this pilot implementation study. Adherence to the counseling protocol was supported through (i) open communication with the program’s lead accredited dietitian, (ii) timely deposition of results to the 6th Health Region Administration representative, and (iii) frequent follow-up reminders from the 6th Health Region Administration.

### 2.2. Intervention Overview

Standardized measures were used within the Smart Family framework to assess lifestyle and behavioral outcomes. The intervention consisted of 4 monthly face-to-face sessions between the trained healthcare professional and the parent–child dyad. For each session, investigators provided healthcare professionals with standardized instructions, guidelines, and tools. Session instructions were developed by the lead accredited dietitian, who had received primary training in the Smart Family methodology from the Finnish Institute for Health and Welfare (THL). Further details are provided in the Supplementary Materials. Consistent with the Smart Family methodology, all sessions emphasized positive interaction between families and healthcare professionals and used a family-centered counseling approach.

During Session 1, child anthropometric measurements, sociodemographic information, and parent and child dietary data were collected using validated food frequency questionnaires (FFQs). The Child Health Card was distributed to the primary guardian at the end of the session to complete at home and was reviewed jointly with the healthcare professional at the start of Session 2.

Session 2 focused on reviewing the Child Health Card responses, establishing family-specific behavioral goals, and discussing children’s weight status using the stigma-free communication approach in which professionals were trained. Parental Mediterranean diet adherence was discussed, and age-appropriate handouts on satiety recognition and food preferences were provided to children, alongside age-specific food group recommendations for parents.

Session 3 introduced the concept of a balanced diet through discussion of the handouts completed since Session 2. Healthcare professionals were provided with targeted tips handouts addressing common questions and challenges reported by families on this topic (examples included in Supplementary Materials Table S1). The use of food as a behavioral reward was addressed, with alternative non-food strategies discussed, including praise, additional physical activity time, and preferred family activities.

Session 4 focused on lifestyle behaviors, including adequate sleep, regular physical activity, and reduced screen time. Family-specific recommendations were provided to support progress towards each dyad’s identified goals.

Each dyad was scheduled to receive four face-to-face sessions at approximately monthly intervals (±1 week) of 20 min (±10) duration. All dyads received the same core Smart Family session structure and materials, while individual goals and counseling emphasis were tailored to the family’s responses on the Child Health Card and discussion with the healthcare professional. Training completion was recorded with stamped dates and on all session documents, and professionals received standardized session instructions and materials for the next session. Formal post-training competency testing and independent fidelity scoring were not conducted in this pilot.

During Sessions 1 and 4, children’s dietary behavior was assessed using a country-specific validated semi-quantitative FFQ containing 34 food items. Items were grouped into major food groups, as described in Supplementary Table S2. The dietary outcome for this pilot analysis was the within-child change in parent-reported frequency of consumption of these food groups between baseline and post-intervention. The FFQ was not used to estimate portion size, total energy intake, or nutrient adequacy, as the aim was to explore changes in reported food group consumption behavior rather than to quantify total dietary intake.

### 2.3. Study Population

The target population included families residing in the 6th Health Region of Greece with at least one child aged 2–12 years, irrespective of weight status. Families were recruited by healthcare professionals trained in the Smart Family methodology in participating primary care units. Recruitment was therefore opportunistic within routine primary care contacts and depended on professional participation and parental consent. Families were excluded if the child had a diagnosed mental health condition, specific dietary guidelines for endocrinological, or other medical condition. Participating families received written study information and provided informed consent before enrollment. In total, 63 parents consented to participate, 50 parent–child dyads enrolled, and 49 dyads completed the program and were included in the final analysis. One dyad was excluded because of missing child information.

### 2.4. Data Collection

#### Anthropometric Measurements (Children)

Children were measured barefoot and wearing light clothing, using standardized anthropometric procedures. Body weight was measured in kilograms and recorded to the nearest 100 g. Height was measured with the child standing upright against a stadiometer, barefoot, with the head positioned in the Frankfurt plane, and was recorded to the nearest 0.1 cm. Weight status was classified using age- and sex-specific WHO growth reference curves (WHO Growth Reference, 2007) [21]. Specifically, Body Mass Index (BMI) for age was classified as follows:

Severe thinness: BMI for age < −3 SD.

Thinness: −3 SD ≥ BMI for age < −2 SD.

Healthy weight: −2 SD ≥ BMI for age < +1 SD (equivalent to BMI < 25 kg/m^2^ at 19 years).

Overweight: +1 SD ≤ BMI for age < +2 SD (equivalent to BMI ≥ 25 kg/m^2^ at 19 years).

Obesity: BMI-for-age ≥ +2 SD (equivalent to BMI ≥ 30 kg/m^2^ at 19 years).

Parents were then asked about their perception of their child’s weight status, diet, and physical activity level. For perceived child weight status, parents classified their child as having lower than normal weight, normal weight, or higher than normal weight. For comparison with parental perception, measured child weight status was collapsed into three categories corresponding to the parental response options: lower weight (severe thinness or thinness), normal weight (healthy weight), and higher weight (overweight or obesity). Parental perception was classified as accurate when the perceived category matched this collapsed measured category, as underestimated when the parent selected a lower category than the measured category, and as overestimated when the parent selected a higher category. Parental perception was then cross-tabulated against the child’s measured weight status based on standardized anthropometric assessment. Parental weight and height were self-reported, and socioeconomic information, including employment status and home ownership, was recorded.

Parents were also asked which food groups they believed their child should increase or decrease. These reported goals were compared descriptively with observed changes in dietary intake between baseline and post-intervention assessment.

### 2.5. Dietary Intake Assessment

During Sessions 1 and 4, children’s dietary intake frequency was assessed using a country-specific validated semi-quantitative food frequency questionnaire containing 34 food items [22]. Items were grouped into nine food groups, as described in Supplementary Table S2. Whole wheat product frequency was derived from the grains and cereals FFQ item and a follow-up question asking how often the consumed grain/cereal products were whole wheat. Response options were coded as follows: “never” = 0%, “sometimes” = 50%, and “always” = 100% of the reported grain/cereal frequency. The resulting whole wheat frequency therefore represents an estimated frequency based on the reported grain/cereal frequency multiplied by the proportion classified as whole wheat. The FFQ was completed by the same parent or primary guardian with the healthcare professional. The FFQ assessed reported frequency of consumption and did not quantify portion size, total energy intake, or nutrient adequacy. Therefore, changes were interpreted as changes in reported consumption frequency rather than changes in total intake. Parental dietary intake was assessed using the main food groups included in the Mediterranean diet, and parental adherence was calculated using the MedDiet score, which ranges from 0 to 55 [23].

### 2.6. Statistical Analysis

The sample size was based on precision for estimating feasibility rather than on statistical power for dietary outcomes. The primary feasibility parameter was completion of the four-session Smart Family counseling protocol among consenting/included dyads. Assuming an expected completion proportion of 80%, 62 dyads would estimate this proportion with a 95% confidence interval half-width of approximately 10 percentage points [n = 1.96^2^ × 0.80 × 0.20/0.10^2^ = 61.5]. Thus, the inclusion of 63 dyads was considered adequate for estimating feasibility with acceptable precision. Dietary outcomes were secondary and were not used for sample size determination. Probability plots were used to assess the distribution of continuous variables. Normally distributed variables are presented as mean and standard deviation, while variables with skewed distributions are presented as median and interquartile range. Categorical variables are presented as counts and percentages. Descriptive analyses were performed overall and by parental MedDiet score tertile. Between-tertile differences were assessed using Pearson’s chi-square test or Fisher’s exact test for categorical variables, and one-way ANOVA or the Kruskal–Wallis test for continuous variables, depending on distributional assumptions. Differences in parental perception of child weight status relative to measured child weight status were examined using cross-tabulations and Pearson’s chi-square or Fisher’s exact test, as appropriate. The Wilcoxon signed-rank test was used to assess within-group changes in food group consumption between baseline and post-intervention. Spearman rank correlations were used to examine associations between continuous MedDiet score and food group change scores. The Mann–Whitney test was used to compare MedDiet scores between parents who underestimated and those who overestimated their child’s weight status. Analyses by parental MedDiet score tertile were exploratory and unadjusted. Because of the small sample size and sparse cells after stratification, these subgroup analyses were used only to generate hypotheses for future controlled studies. No adjustment for multiple comparisons was applied. All *p*-values should therefore be interpreted as nominal and exploratory, consistent with the pilot and hypothesis-generating purpose of the study. Statistical significance was set at α = 0.05 using two-tailed tests. Analyses were performed using Stata 18.0. (StataCorp LLC, College Station, TX, USA).

## 3. Results

### 3.1. Feasibility Indicators

Of the 14 healthcare professionals who completed Smart Family training, eight participated in the implementation phase. Sixty-three parents consented to participate, 50 parent–child dyads enrolled in the program, and 49 dyads completed the four-session intervention and were included in the final analysis. This corresponded to an analytic completion rate of 98.0% among enrolled dyads. The intervention was delivered as four monthly face-to-face counseling sessions in the primary care setting. Baseline and post-intervention dietary data were available for all dyads included in the analytic sample.

Of the 63 included parent–child dyads, 49 completed the Smart Family intervention (79% completion); most usual reason was “lack of time” by parents. Of the 50 dyads enrolled and commenced, all attended all four counseling sessions. One dyad had missing outcome information; because missingness was minimal, no imputation was performed. Feedback from trained healthcare professionals at the end of the intervention through a valid questionnaire (HSDQ) indicated general acceptance of the program. The main implementation concern was the time required to deliver the intervention alongside routine duties and the fast pace of work in health centers.

### 3.2. Participant Characteristics

Baseline characteristics of the 49 parent–child dyads included in the final analysis are presented in Table 1, stratified by parental MedDiet score tertile. Participating children had a mean age of 7.0 years (SD 2.7), with 55.1% boys and 44.9% girls. Based on WHO growth reference criteria, 71.4% of children had normal weight, 14.3% had overweight, 6.1% had obesity, and 6.1% had thinness. Of the 49 participating parents, 48 were mothers and one was a father. Mean parental MedDiet score was 28.9 (SD 3.7), with 17 dyads (34.7%) in the first tertile (mean score 24.9, SD 1.9), 20 dyads (40.8%) in the second tertile (mean 29.6, SD 1.2), and 12 dyads (24.5%) in the third tertile (mean 33.6, SD 1.8). Maternal age differed significantly across tertiles (*p* = 0.003), with mothers in the second tertile being of older age (mean 42.8, SD 3.7 years) than those in the first (mean 38.1, SD 3.7 years) and third tertiles (mean 39.8, SD 4.8 years). The distribution of children’s measured weight status did not differ significantly across MedDiet score tertiles. However, the proportion of children with overweight or obesity was descriptively higher in the third tertile (41.7%) than in the first (17.6%) and second tertiles (10.0%). No statistically significant between-tertile difference in measured child weight status was observed.

**Figure 1:**
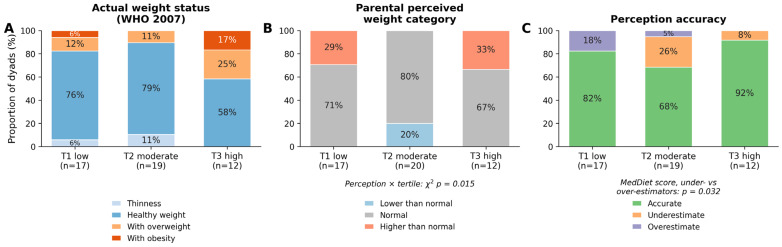
Child weight status, parental perception, and perception accuracy by parental MedDiet score tertile. Panel (**A**) shows the distribution of actual child weight status classified by the WHO 2007 growth reference; panel (**B**) shows the distribution of the parental perceived weight category; and panel (**C**) shows perception accuracy, classified as accurate, underestimated, or overestimated relative to measured status. Each column represents one MedDiet score tertile: T1 low adherence (n = 17, mean score 24.9), T2 moderate adherence (n = 19, mean score 29.6), and T3 high adherence (n = 12, mean score 33.6). Within each column, the upper panel shows the actual distribution of child weight status according to WHO growth reference criteria (2007); the middle panel shows the distribution of parental perceived child weight category (lower, normal, or higher weight); and the lower panel shows the proportion of parents whose perception was accurate, underestimated (perceived lighter than actual), or overestimated (perceived heavier than actual). Perception distribution across tertiles was assessed using Pearson’s chi-square (*p* = 0.015). The Mann–Whitney test comparing MedDiet scores between parents who underestimated and parents who overestimated their child’s weight status was significant (*p* = 0.032). Statistical significance was set at alpha = 0.05.

Figure 1 depicts that the distribution of parental perceived weight category differed significantly across MedDiet score tertiles (*p* = 0.015). In the first tertile (low adherence, n = 17), no parent underestimated their child’s weight status; however, 17.6% overestimated it, perceiving their child as heavier than indicated by measured weight status. All three children with overweight or obesity in this tertile were correctly identified as having higher weight status. In the second tertile (moderate adherence, n = 19), 21.1% of parents underestimated their child’s weight status, perceiving them as thinner than measured, while one parent (5.3%) overestimated. In the third tertile (high adherence, n = 12), 91.7% of parents were accurate; 8.3% underestimated and none overestimated. When MedDiet score was examined as a continuous variable, parents who underestimated their child’s weight status had significantly higher MedDiet scores than parents who overestimated (*p* = 0.032). Given the small expected cell frequencies, these findings should be interpreted as exploratory.

Table 2 presents the proportion of parents reporting food groups they wished their child to increase or decrease during the intervention. Sugar-sweetened beverages were the most frequently reported food group targeted for reduction (91.8%), followed by sweets and desserts (77.6%) and grains and cereals (32.7%).

### 3.3. Changes in Dietary Intake Frequency from Baseline to Post-Intervention

Table 3 presents changes in parent-reported frequency of children’s consumption of specific food groups between baseline and post-intervention. The largest reduction was observed for sweets and desserts, with a median decrease of 2.35 servings per week (IQR −4.15 to 0.00, *p* < 0.001). Statistically significant within-group changes were also observed for sugar-sweetened beverages, total grains and cereals, whole wheat products, and dairy intake. Fish consumption increased significantly, and a small but statistically significant increase was observed for vegetable intake. Despite 59.2% of parents reporting fruit as a target for increase and 79.6% reporting vegetables as a target for increase, fruit intake did not change significantly, and the increase in vegetable intake was modest. Reported dairy frequency also decreased significantly, although the change was small (max frequency decrease −0.64 per day; *p* = 0.008). No statistically significant within-group changes were observed for fast food, red meat, legumes, or white meat in the total sample.

When dietary change scores were examined by parental MedDiet score tertile, statistically significant between-tertile differences were observed for fast food and red meat (Table 4). For fast food consumption, children in the first tertile showed a median reduction of 0.65 servings per week (IQR −0.95 to 0.00), children in the second tertile showed a median reduction of 0.50 servings per week (IQR −1.09 to 0.35), and children in the third tertile showed a median increase of 0.32 servings per week (IQR 0.00 to 1.39). For red meat consumption, a within-tertile reduction was observed only in the first tertile (*p* = 0.024), with no statistically significant change in the second or third tertiles. For all other food groups, change scores did not differ significantly across MedDiet score tertiles. Given the small number of participants in each tertile, these stratified findings should be interpreted as exploratory.

## 4. Discussion

This single-arm pilot study examined the implementation of the Smart Family counseling methodology in Greek primary care as part of the Health4EUkids Joint Action. Over four months, the intervention was feasible to deliver through trained healthcare professionals and was followed by reductions in selected discretionary foods, particularly sweets and desserts. In contrast, changes in fruit and vegetable intake were limited despite these being common parental goals. The reduction in reported dairy frequency requires caution. Unlike reductions in sweets and sugar-sweetened beverages, lower dairy frequency cannot be assumed to be beneficial in children. The FFQ-based dairy variable captured the frequency of dairy consumption but did not quantify portion size, distinguish adequately between plain and sweetened/flavored dairy products for nutritional interpretation, or assess replacement foods. Therefore, the observed decrease may reflect several possibilities, including reduced sweetened dairy intake, reduced plain dairy intake, reporting variation, or dietary substitution. Because dairy products may contribute calcium, protein, and other micronutrients in children’s diets, this finding should be interpreted as nutritionally uncertain and should be examined more precisely in future studies. Exploratory analyses suggested that parental perception of child weight status and parental Mediterranean diet adherence may be relevant to dietary change patterns, although these findings require confirmation in larger controlled studies.

Evidence from longer individualized family interventions, including the STRIP study, indicates that durable change in children’s dietary patterns and weight-related outcomes requires sustained exposure over years rather than months [13,16]. Against this background, the reductions in sweets and sugar-sweetened beverages observed in the present study are consistent with the feasibility of targeting these specific dietary behaviors within a four-month counseling period, although they cannot be attributed causally to the intervention. The limited response in fruit and vegetable intake further underlines the difficulty of changing habit-bound behaviors over this timeframe. Reducing a specific unhealthy item is a clearly bounded behavior that parents can act upon immediately and is often the first concrete action in family counseling; increasing vegetables, by contrast, requires changes in purchasing, preparation and the management of child food neophobia, a well-recognized developmental phenomenon that typically requires repeated exposures and sustained parental persistence to overcome [24]. The present study is therefore best interpreted as a feasibility and preliminary outcomes signal indicating that the Smart Family counseling methodology can be delivered as planned in a Greek primary care context and is associated with short-term changes in selected discretionary foods, while durable changes in children’s food environments are likely to require longer and more intensive intervention evaluated in controlled design.

A central observation of this pilot concerns parental perception of child weight status, which may be important for intervention engagement. If counseling begins with a parent who does not recognize that their child carries excess weight, or who perceives a healthy-weight child as overweight, the motivational basis of the intervention may be compromised before any dietary advice is offered. In this sample, parents with low MedDiet adherence tended to overestimate their child’s weight status, whereas parents with moderate adherence tended to underestimate it, with children with overweight in the moderate tertile perceived as having normal weight; parents in the highest adherence tertile showed the highest apparent accuracy, although this tertile also contained the highest proportion of children with overweight or with obesity, which limits interpretation. Underestimation of child weight has been associated with greater parental optimism about a child’s diet quality and physical activity, which may reduce receptiveness to counseling [9], and is common at the population level, with the WHO COSI initiative reporting that 82.3% of parents of children with overweight underestimated their child’s weight status across 22 countries, and higher rates still among parents of children with obesity [25]. Parents may also overestimate the quality of their child’s diet despite intending to provide a healthy diet [26]. A narrative review similarly identified misconceptions about diet quality as an important target for early intervention [27]. Taken together, these observations indicate that parental perception of both weight and diet is relevant to how families may engage with family-based counseling.

A further exploratory observation concerns the differences in fast food and red meat change scores across parental MedDiet adherence tertiles with the smallest reductions among families whose parents reported higher adherence and, unexpectedly, the highest proportion of children with overweight or obesity in the highest adherence tertile. We interpret this as a hypothesis-generating observation rather than as evidence of a direct relationship between parental Mediterranean diet adherence and child weight status, and several explanations are possible that the present pilot cannot adjudicate. The MedDiet score was parent-reported, so parents in the highest adherence tertile may have over-reported healthy eating, and apparent adherence may partly reflect social desirability rather than measured dietary quality. The pattern may also reflect a reverse pathway, in which families with a child of higher weight status are more motivated to adopt, or to report, a healthier dietary pattern. A third possibility is that parents who perceive their household diet as already adequate may feel less need to modify specific child behaviors and may therefore be less receptive to counseling messages targeting change, which could help to explain why reductions in selected food groups were not greater in the higher adherence tertile. A simple ceiling effect, of the kind reported in adults in the PREDIMED-Plus trial [28], is not supported here, because baseline fast food and red meat intakes did not differ significantly between tertiles. These interpretations are consistent with evidence that parental perceptions of child weight and diet are related to feeding attitudes, perceived need for change, and children’s dietary behaviors [11,26,27], with findings from the Greek EYZHN study that a combined Mediterranean lifestyle index incorporating diet, physical activity, sedentary behavior, and sleep is inversely associated with childhood overweight and obesity, indicating that dietary adherence is only one component of the lifestyle pattern relevant to weight status [29], and with the Feel4Diabetes finding that the home food environment, parenting practices, and parental health beliefs are associated with the weight status of European children [30]. Within the Smart Family framework, counseling may therefore need to screen for parental dietary and weight status perception, rather than emphasizing only the food groups that families themselves nominate for change, and to set concrete behavior-specific targets together with families in a non-stigmatizing way, particularly when parents already view the household diet as healthy; adapting counseling content to each parent’s perception in this way may be more effective than generic messaging [31]. This remains a hypothesis for future implementation research, but it is directly relevant to adapting structured family-centered counseling so that parental perceived adequacy does not reduce engagement with specific dietary goals. The present pilot explored changes in reported child dietary behaviors rather than weight, portion size, or energy intake, and future studies incorporating child diet quality, physical activity, sleep, sedentary behavior, and family feeding practices are needed to clarify how parental dietary patterns relate to child weight status.

This study has several strengths relevant to feasibility and implementation. The intervention was based on the Smart Family approach, a European best practice that emphasizes positive family role modeling rather than prescriptive restriction and was delivered through face-to-face counseling by trained healthcare professionals in a primary care setting. Combining measured anthropometric data with parental perception of child weight and with parental dietary quality allowed us to identify a perception pattern that would have remained invisible had we examined dietary change alone.

These findings should be interpreted in light of several limitations. Recruitment and refusal rates, number approached, reasons for refusal, and population reach were not systematically recorded. In addition, acceptability and fidelity were not assessed using formal validated instruments. However, implementation feedback from trained healthcare professionals was collected, and protocol adherence was supported through structured communication and follow-up procedures during delivery. Parental height and weight were self-reported, which may have introduced reporting bias and potential misclassification of parental BMI. However, parental BMI was collected only for descriptive characterization of the study sample and was not used in the interpretation of feasibility outcomes, dietary change outcomes, or parental perception findings. Because this was a single-arm pretest–posttest pilot study without a concurrent control group, observed changes cannot be attributed causally to the intervention. Seasonal variation, repeated completion of the FFQ, increased awareness of target behaviors, differential social desirability after counseling, and baseline variability may have contributed to the observed changes. The four-month follow-up period was short relative to the time course over which BMI-for-age z-scores would be expected to change meaningfully; therefore, anthropometric change was not assessed as a primary endpoint, and the analysis focused on dietary consumption frequency rather than portion size, energy intake, or weight change. The observed differences across MedDiet score tertiles should be interpreted cautiously. They may identify issues for future investigation, but the present study was not powered to determine whether parental Mediterranean diet adherence or weight status perception modifies dietary response to counseling. Dietary outcomes were based on parent-reported FFQ data and should therefore be interpreted as reported food group consumption frequencies rather than estimates of portion size, total energy intake, or nutritional adequacy. Recall error and social desirability bias may have affected reporting, and differential reporting at follow-up is possible because the intervention explicitly addressed healthy eating behaviors. These measurement characteristics limit nutritional and quantitative intake inference, but they are consistent with the study’s behavioral aim. Also, because eight healthcare professionals delivered the intervention, responses may have been partially clustered by provider; however, the small sample and limited number of dyads per professional precluded reliable multilevel modeling. Larger controlled studies with longer follow-up, incorporating physical activity measurement and repeated assessment of parental perception, are needed to test whether the preliminary signals reported here translate into durable improvements in children’s diet quality and weight status. Future adaptations of the intervention should more directly address the intention–behavior gap, particularly where parents recognize the value of increasing fruit and vegetable intake but lack practical strategies to overcome barriers such as child refusal, preparation time, cost, or limited availability.

## 5. Conclusions

This single-arm pilot study suggests that the Smart Family counseling methodology can be implemented in Greek primary care over four months with a high session completion rate among enrolled dyads. Reductions in selected discretionary foods were observed alongside the intervention, but cannot be attributed causally to the intervention in the absence of a concurrent control group. However, changes in fruit and vegetable intake were limited despite being common parental goals. Parental perception of child weight status and parental Mediterranean diet adherence appeared to be relevant to dietary change patterns, but these findings are exploratory and require confirmation in larger controlled studies. Future family-based obesity prevention interventions should incorporate strategies that address parental weight perception, portion size, discretionary food intake, and the practical barriers that limit translation of dietary intentions into sustained behavior change.

## Figures and Tables

**Table 1:** Baseline characteristics of study participants by Mediterranean diet score tertile (n = 49).

Variable	T1 Low Adherence (n = 17)	T2 Moderate Adherence (n = 20)	T3 High Adherence (n = 12)	Total (n = 49)	*p*-Value
**MedDiet score, mean (SD)**	24.9 (1.9)	29.6 (1.2)	33.6 (1.8)	28.9 (3.7)	<0.001
Maternal age, mean (SD)	38.1 (3.7)	42.8 (3.7)	39.8 (4.8)	40.4 (4.4)	0.003
Paternal age, mean (SD)	42.4 (4.3)	44.5 (5.2)	41.9 (3.6)	43.1 (4.6)	0.208
**Home owned by family, n (%)**					0.876
Yes	12 (70.6%)	15 (75.0%)	8 (66.7%)	35 (71.4%)	
No	5 (29.4%)	5 (25.0%)	4 (33.3%)	14 (28.6%)	
Satisfaction with paternal income (1–5), mean (SD)	3.5 (0.6)	3.4 (0.6)	3.0 (0.4)	3.3 (0.6)	0.081
Satisfaction with maternal income (1–5), mean (SD)	2.6 (1.0)	3.0 (0.9)	2.6 (1.2)	2.7 (1.0)	0.463
**Maternal weight status, n (%)**					0.165
Healthy weight	13 (76.5%)	12 (60.0%)	4 (33.3%)	29 (59.2%)	
With overweight	3 (17.6%)	3 (15.0%)	4 (33.3%)	10 (20.4%)	
With obesity	1 (5.9%)	5 (25.0%)	4 (33.3%)	10 (20.4%)	
**Paternal weight status, n (%)**					0.639
Healthy weight	3 (17.6%)	6 (30.0%)	3 (25.0%)	12 (24.5%)	
With overweight	9 (52.9%)	7 (35.0%)	7 (58.3%)	23 (46.9%)	
With obesity	5 (29.4%)	7 (35.0%)	2 (16.7%)	14 (28.6%)	
**Child’s weight status—parental perception, n (%)**					0.015 *
Lower weight status	0 (0.0%)	4 (20.0%)	0 (0.0%)	4 (8.2%)	
Normal weight status	12 (70.6%)	16 (80.0%)	8 (66.7%)	36 (73.5%)	
Higher weight status	5 (29.4%)	0 (0.0%)	4 (33.3%)	9 (18.4%)	
Parental perception of child’s diet (1–10), mean (SD)	7.4 (1.3)	7.5 (1.3)	6.5 (2.5)	7.2 (1.7)	0.538
**Parental perception of child’s diet—categorized, n (%)**					0.266
1–3 (poor)	0 (0.0%)	0 (0.0%)	1 (9.1%)	1 (2.1%)	
4–7 (moderate)	10 (58.8%)	9 (45.0%)	7 (63.6%)	26 (54.2%)	
8–10 (good)	7 (41.2%)	11 (55.0%)	3 (27.3%)	21 (43.8%)	
**Meal family eats together, n (%)**					0.071
Breakfast	0 (0.0%)	4 (20.0%)	0 (0.0%)	4 (8.2%)	
Lunch	8 (47.1%)	10 (50.0%)	4 (33.3%)	22 (44.9%)	
Supper	9 (52.9%)	6 (30.0%)	8 (66.7%)	23 (46.9%)	
**Frequency of eating/ordering out, n (%)**					0.168
1–2 times/month	10 (58.8%)	13 (65.0%)	8 (66.7%)	31 (63.3%)	
3–4 times/month	7 (41.2%)	6 (30.0%)	1 (8.3%)	14 (28.6%)	
2 times/week	0 (0.0%)	1 (5.0%)	2 (16.7%)	3 (6.1%)	
≥3 times/week	0 (0.0%)	0 (0.0%)	1 (8.3%)	1 (2.0%)	
**Child’s physical activity—parental perception, n (%)**					0.537
Very low–low	2 (11.8%)	4 (20.0%)	2 (16.7%)	8 (16.3%)	
Adequate	13 (76.5%)	13 (65.0%)	6 (50.0%)	32 (65.3%)	
Intense–very intense	2 (11.8%)	3 (15.0%)	4 (33.3%)	9 (18.4%)	
**Family active together, n (%)**					0.429
Never	7 (43.8%)	9 (52.9%)	3 (33.3%)	19 (45.2%)	
Sometimes	7 (43.8%)	7 (41.2%)	3 (33.3%)	17 (40.5%)	
Often	2 (12.5%)	1 (5.9%)	3 (33.3%)	6 (14.3%)	
**Child weight status (WHO), n (%)**					0.395
Thinness	1 (5.9%)	2 (10.0%)	0 (0.0%)	3 (6.1%)	
Normal weight	13 (76.5%)	15 (75.0%)	7 (58.3%)	35 (71.4%)	
With overweight	2 (11.8%)	2 (10.0%)	3 (25.0%)	7 (14.3%)	
With obesity	1 (5.9%)	1 (5.0%)	2 (16.7%)	4 (8.2%)	

Fisher’s exact test was used where expected cell counts were ≤5. * significant at *p* < 0.05.

**Table 2:** Percent parents reporting foods they would like their child to increase or decrease intake.

Food Group	Increase	Decrease
N	50	
Grains and cereals	1 (2.0%)	16 (32.7%)
Dairy	12 (24.5%)	1 (2%)
Sweets	1 (2%)	38 (77.6%)
Sodas	0	45 (91.8%)
Juice	7 (14.3%)	4 (8.2%)
Fats & oils	1 (2.0%)	10 (20.4%)
Red meat	12 (24.5%)	3 (6.1%)
Vegetables	39 (79.6%)	0
Fruit	29 (59.2%)	0

**Table 3:** Reported child intake of specific food groups at baseline and post-intervention.

Food Group	Unit	Baseline Median (IQR)	Post-Intervention Median (IQR)	Change Median (IQR)	*p*-Value
Grains & Cereals	weekly	1.64 (1.43, 2.07)	1.44 (1.14, 1.86)	−0.28 (−0.42, −0.05)	0.001 **
Whole Wheat Products	weekly	2.00 (2.00, 3.00) †	1.35 (0.44, 1.35)	−1.30 (−1.91, −0.77)	<0.001 ***
Vegetables	daily	0.79 (0.29, 1.00)	0.79 (0.43, 1.14)	+0.09 (0.00, 0.29)	0.026 *
Fruit	weekly	7.00 (4.50, 7.00)	7.00 (4.50, 7.00)	0.00 (0.00, 0.00)	0.923
Legumes	weekly	1.00 (1.00, 2.00)	1.00 (1.00, 2.00)	0.00 (0.00, 0.00)	0.486
Dairy	daily	1.64 (1.29, 2.00)	1.29 (0.93, 2.00)	0.00 (−0.64, 0.00)	0.008 **
Red Meat	weekly	2.00 (2.00, 2.00)	2.00 (1.00, 2.00)	0.00 (0.00, 0.00)	0.090
White Meat	weekly	2.00 (1.00, 2.00)	2.00 (1.00, 2.00)	0.00 (0.00, 0.00)	0.520
Fish	weekly	1.00 (1.00, 1.00)	1.00 (1.00, 2.00)	0.00 (0.00, 1.00)	0.005 **
Sweets & Desserts	weekly	8.85 (6.00, 14.50)	6.35 (3.70, 10.85)	−2.35 (−4.15, 0.00)	<0.001 ***
Sugar-Sweetened Beverages	weekly	1.00 (0.00, 2.70)	0.64 (0.00, 2.14)	0.00 (−1.60, 0.00)	0.017 *
Fast Food	weekly	2.70 (1.75, 4.40)	2.40 (1.75, 3.40)	−0.30 (−1.00, 0.65)	0.301
Oils & Fats	weekly	7.00 (7.00, 7.70)	7.00 (7.00, 7.70)	0.00 (−0.65, 0.22)	0.955

Data presented as median (interquartile range). *p*-values from Wilcoxon signed-rank tests comparing baseline (Session 1) and post-intervention (Session 4). † Whole wheat products: baseline value is the aggregated weekly serving total: reported grains and cereals frequency multiplied by the reported proportion consumed as whole wheat (“never” = 0%, “sometimes” = 50%, “always” = 100%); post-intervention data were computed via change in frequency of consumption: IQR: interquartile range. * *p* < 0.05; ** *p* < 0.01; *** *p* < 0.001.

**Table 4:** Food group change scores by parental MedDiet score tertile.

Food Group	Unit	T1 Low Median (IQR)	* p * (T1)	T2 Moderate Median (IQR)	* p * (T2)	T3 High Median (IQR)	* p * (T3)	KW *p*
Grains & Cereals	weekly	0.00 (−2.50–2.00)	0.861	0.00 (−2.50–0.09)	0.196	0.32 (−0.41–2.50)	0.164	0.271
Whole Wheat Products	weekly	−1.35 (−2.00–−0.70)	0.001 **	−1.30 (−2.00–−0.92)	0.001 **	−1.15 (−1.65–−0.91)	0.001 **	0.633
Vegetables	weekly	0.65 (0.00–2.30)	0.080	1.25 (0.00–2.50)	0.034 *	0.00 (−1.38–1.00)	0.688	0.172
Fruit	weekly	0.00 (0.00–0.00)	0.196	0.00 (0.00–0.00)	1.000	0.00 (0.00–1.04)	—	0.325
Legumes	weekly	0.00 (0.00–0.35)	0.620	0.00 (0.00–0.00)	0.216	0.00 (−0.65–0.00)	0.688	0.396
Dairy	daily	0.00 (0.00–0.00)	0.398	0.00 (−0.77–0.11)	0.132	−0.18 (−0.86–0.00)	0.047 *	0.466
Red Meat	weekly	0.00 (−1.00–0.00)	0.024 *	0.00 (0.00–0.00)	0.414	0.00 (0.00–0.00)	—	0.047 *
White Meat	weekly	0.00 (0.00–0.00)	0.527	0.00 (0.00–0.25)	0.831	0.00 (0.00–0.25)	—	0.970
Fish	weekly	0.00 (0.00–1.00)	0.062	0.00 (0.00–0.25)	0.025 *	0.00 (0.00–0.65)	0.281	0.887
Sweets & Desserts	weekly	−1.65 (−4.15–−0.05)	0.011 *	−2.50 (−3.50–−0.75)	0.003 **	−1.68 (−5.55–1.38)	0.182	0.936
Sugar-Sweetened Beverages	weekly	−1.00 (−1.65–0.30)	0.177	0.00 (−0.78–0.42)	0.975	0.00 (−0.52–0.00)	0.625	0.570
Fast Food	weekly	−0.65 (−0.95–0.00)	0.086	−0.50 (−1.09–0.35)	0.092	0.32 (0.00–1.39)	0.156	0.022 *

Data presented as median change (IQR) between baseline and post-intervention. *p* (T1/T2/T3): within-tertile Wilcoxon signed-rank test. KW *p*: Kruskal–Wallis test for difference in change scores across tertiles. Given the small tertile sample sizes (n = 17, 20, 12), these analyses are exploratory and should not be interpreted as confirmatory. — indicates insufficient non-zero differences for the Wilcoxon test. * *p* < 0.05; ** *p* < 0.01. T1: low adherence; T2: moderate adherence; T3: high adherence.

## Data Availability

The data presented in this study are available on request from the corresponding author due to privacy considerations relating to participant anonymity.

## Supplementary Materials

The following supporting information can be downloaded at: https://www.mdpi.com/article/10.3390/nu18121848/s1, Table S1: information and activities per Smart Family methodology Greek pilot intervention session; Table S2: FFQ food categorization into nine food groups; tips for families to be provided by trained healthcare professionals as needed.
