# Supplementary material for: Feasibility and Preliminary Dietary Outcomes of the Smart Family Lifestyle Counseling Intervention in Greek Primary Care: A Single-Arm Pilot Study from Health4Eukids
Source: Nutrients. 2026 Jun 8;18(12):1848. doi: 10.3390/nu18121848 (PMC13304528; doi:10.3390/nu18121848)
Supplement: Supplementary file 1 [file nutrients-18-01848-s001.zip › nutrients-4348632-supplementary.pdf]

Supplementary material

STable1. Information and Activities per Smart Family methodology Greek Pilot Intervention Session.

Session	Parental Info	Children's Info & Activities	Joint Family Activities
Session 1	Completion of Parental Questionnaire<br>+<br>Validated child FFQ<br>Provide and explain Child Health Card	Anthropometric measurements of weight and height	Families establish up to three achievable behavioral goals
Session 2: Set specific nutrition and lifestyle goals to achieve – Discuss children's weight status avoiding stigma and introduce healthy nutrition through family's intake example	Review Child Health Card and set initial goals<br><br>Food Group Recommendations and age specific portion sizes handout	BMI calculated discussed using Smart Family principles (avoiding stigma)<br><br>Satiety-hunger assessment through drawing; The Happy Child; My Food Preferences	Discuss Child Health Card and set goals (up to 3) for next session<br>→Reinforce positive practices<br><br>Calculate and discuss Mediterranean Diet adherence and provide example based on family's intake
Session 3: Nutrition Oriented on an age specific balanced diet/plate –<br>Discuss Good Behavior ≠ Bad habits rewarding avoidance	Review and discuss completed child handouts<br>Provide rewarding ideas to avoid reinforcing unhealthy behaviors	Handouts on screen, sleep and physical activity (one each)	Emphasis on fruits and vegetables and methods in adding new foods
Session 4: Lifestyle Oriented in relation to Nutrition + Follow up assessments	Repeat child FFQ<br><br>Discuss handouts on sleep, screen time, and physical activity as per requirements and set goals.	Anthropometric measurements of weight and height	Place goals for sleep, screen, physical activity discussed in this session.<br><br>Reinforce parental skills for methods to achieve goals and continuation of effort.

FFQ: Food Frequency questionnaire; Anthropometric measurements included weight, height and waist circumference; Smart Family principles included specific wording for feedback based on children's weight status categorization; Mediterranean Diet adherence as per MedDietScore, adapted for children.

Stable2. FFQ food categorization into eleven food groups

**Food Groups**	
Grains and Cereals	Breakfast cereals (plain cornflakes or with chocolate or fruit)
	Pasta
	Bread (including toast), pita bread
Dairy	Cheese (e.g., feta or gouda)
	Milk or plain yogurt
	Milk or yogurt with cocoa or chocolate or fruit
Sweets	Chocolate or wafer
	Chocolate spread
	Ice cream
	Cake or croissant
	Jam or honey
	Biscuit or cereal bar
Sodas	Soft drink
	Light soft drink
Juice	100% natural juice
	Nectar or fruit juice
Fats & Oils	Margarine
	Mayonnaise or ready-made sauce
	Olive oil
Red meat & Animal protein	Red meat (beef or pork) or cold cuts (salami, ham)
	White meat (chicken or turkey)
	Fish
Vegetables	Raw vegetables (e.g., cabbage, carrots, tomatoes, cucumbers)
	Boiled vegetables (e.g., green beans, broccoli, greens)

Fruit	Fruit (apple, banana, mandarins, etc.)
Pulses	Beans or lentils or chickpeas or fava beans
Fast food	Cheese pie, spinach pie, or bougatsa
	French fries
	Potato chips or popcorn or pretzels
	Pizza
	Hamburger
	Gyros or souvlaki in pita bread or bread

Pulses, fats, and oils were not used for the current analysis.

Tips for Families to be provided by trained Health care professionals as needed

LEARNING ABOUT VEGETABLES:

- *Serve vegetables to children in a form that they find interesting and in recognisable pieces.*
- *Use colour theme days to encourage children to familiarise themselves with vegetables of different colours.*
- *Let the children choose themselves the products that they like in the fruit and vegetable section of the store.*
- *Let the children participate in the preparation of salads and grated vegetables.*
- *Favour vegetables of different colours, both fresh and cooked, because different vegetables contain different beneficial compounds. Boiled potatoes are a good choice for a side dish.*
- *By choosing vegetables and berries that are in season you will get the best culinary experiences, be environmental responsible and save money.*

INCREASE VARIETY OF MAIN MEALS WITH VEGETABLES:

- *Supplement warm foods with vegetables.*
- *Add vegetarian foods on the weekly menu.*
- *Choose vegetables and vegetable spreads for sandwich toppings.*
- *Meat can be replaced with various bean, pea, broad bean, seed and soy products (ie tofu), as well as lentils.*
- *Favour fish, chicken and vegetarian foods.*
- *Cut back on eating foods made from sausages and fatty meat and prepare food from low-fat meat products, such as lean (<10%) ground beef.*
- *Choose low-fat meat and deli meats: chicken, turkey, ham and other whole meat products (deli meats fat content max. 4% and salt max. 2%).*

INCREASING FIBRE INTAKE:

- *The best sources of fibre include whole grain products, such as whole grain bread, meals, muesli, and breakfast cereals.*
- *Choose a high-fibre bread with a fibre content of at least 6 g / 100 g.*
- *Instead of white rice, favour pearled grain as well as whole and broken groats, e.g. whole grain rice, a mixture of rye, oats, barley, and rice as well as barley and spelt.*
- *Oven-cooked barley porridge is an easy and tasty alternative for the second main meal of the day.*

- *There are also other good whole grain products in addition to the traditional grains, such as buckwheat, millet, quinoa bulgur and amaranth.*

BEVERAGE CHOICES:

- *Water is the best primary beverage.*
- *Consume fluids at the same intervals as food.*
- *Beverages that contain sugar are the most important source of hidden sugar (**show infographic**)*
- *Choose fruit and berries as such over juices; they have a higher nutritional content than juices.*
- *Limit your consumption of sugar-containing drinks to mealtimes to avoid unnecessary damage to your teeth.*

CUTTING BACK ON SUGAR:

- *Milk products may contain a surprisingly large amount of added sugar. Choose plain yogurt, quark and other fermented milk products ("kefir") and flavour them yourself with berries and fruit.*
- *The sugar content of muesli and breakfast cereals can be more than 30% (e.g. chocolate cereals, muesli cereals, honey cereals) and they are often also high in salt and fat. Choose products with a sugar content of at most 16 g / 100 g.*
- *Favour porridge and unsweetened or homemade muesli for breakfast.*
- *Fruit and berries are acceptable treats every day.*
- *Cookies and biscuits should not be part of the daily diet. They can be replaced with e.g. pieces of root vegetables and fruit, vegetables, berries, banana chips, plain nuts and almonds.*
- *Consider package sizes: a smaller package is enough to satisfy the craving for treats.*
- *Buy smaller bags of sweets instead of the jumbo bags. Choose the suitable package size already in the store. You usually finish the whole bag in any case. A 200-gram bag of sweets, for example, is enough to share between several persons.*
- *Curb your sweet tooth by drawing up a plan that best suits you. For some the best plan is to eat sweets on one day a week, for others a small daily treat is a better alternative.*

CUTTING BACK ON SALT:

- *Use as little salt as possible when preparing food for your family. Measure salt with a spice spoon or a teaspoon, for example, to ensure you use a proper amount of salt.*
- *Salt can be replaced in cooking with herbs, citrus juice (e.g. lemon juice) and vinegar.*
- *Choose low-salt food products in the store.*
- *Do not add salt in the cooking water of e.g. potatoes, vegetables, rice and pasta.*
- *Pay particular attention to the salt content of bread, cheese and deli meats.*
- *Exchange potato crisps for low-salt popcorn.*
